# Toward Exempting from Sentinel Lymph Node Biopsy in T1 Breast Cancer Patients: A Retrospective Study

**DOI:** 10.3389/fsurg.2022.890554

**Published:** 2022-06-28

**Authors:** Guozheng Li, Jiyun Zhao, Xingda Zhang, Xin Ma, Hui Li, Yihai Chen, Lei Zhang, Xin Zhang, Jiale Wu, Xinheng Wang, Yan Zhang, Shouping Xu

**Affiliations:** ^1^ Department ofs Breast Surgery, Harbin Medical University Cancer Hospital, Harbin, China; ^2^ School of Life Science and Technology, Computational Biology Research Center, Harbin Institute of Technology, Harbin, China

**Keywords:** T1 breast cancer, SLNB, exempting, axillary surgery, molecular subtypes

## Abstract

**Background and Objective:**

Sentinel lymph node biopsy (SLNB) is used to assess the status of axillary lymph node (ALN), but it causes many adverse reactions. Considering the low rate of sentinel lymph node (SLN) metastasis in T1 breast cancer, this study aims to identify the characteristics of T1 breast cancer without SLN metastasis and to select T1 breast cancer patients who avoid SLNB through constructing a nomogram.

**Methods:**

A total of 1,619 T1 breast cancer patients with SLNB in our hospital were enrolled in this study. Through univariate and multivariate logistic regression analysis, we analyzed the tumor anatomical and clinicopathological factors and constructed the Heilongjiang Medical University (HMU) nomogram. We selected the patients exempt from SLNB by using the nomogram.

**Results:**

In the training cohort of 1,000 cases, the SLN metastasis rate was 23.8%. Tumor volume, swollen axillary lymph nodes, pathological types, and molecular subtypes were found to be independent predictors for SLN metastasis in multivariate regression analysis. Distance from nipple or surface and position of tumor have no effect on SLN metastasis. A regression model based on the results of the multivariate analysis was developed to predict the risk of SLN metastasis, indicating an AUC of 0.798. It showed excellent diagnostic performance (AUC = 0.773) in the validation cohort.

**Conclusion:**

The HMU nomogram for predicting SLN metastasis incorporates four variables, including tumor volume, swollen axillary lymph nodes, pathological types, and molecular subtypes. The SLN metastasis rates of intraductal carcinoma and HER2 enriched are 2.05% and 6.67%. These patients could be included in trials investigating the SLNB exemption.

## Introduction

Breast cancer has the highest incidence rate among female malignant tumors, accounting for 24.2% of all new cases each year (1). Breast cancer treatment drugs are constantly evolving, as is the concept of surgery. From the initial “expanded radical treatment” to “modified radical treatment,” and to the current “breast-conserving surgery,” all of them reflect that breast cancer surgery focuses not only on effective treatment, but also on maximizing aesthetics and minimizing trauma.

SLN is the first regional lymph node from the primary tumor metastasis and the first lymph node capable of receiving lymph fluid from a specific organ and region (2). It can be used as a treatment and prognostic factor for breast cancers (3–5). Therefore, SLNB can predict the metastasis status of ALNs with a low false-negative rate, allowing more patients to avoid upper limb pain, sensory loss, and lymphedema caused by axillary lymph node dissection (6, 7). However, approximately 65%–70% of patients have suffered from unnecessary invasive axilla surgery (8, 9). This raises the question of whether we can pinpoint who might avoid SLNB.

Several studies have found a strong association between the molecular subtypes and the axillary status in breast cancer patients (10, 11). Furthermore, whether SLNB should be performed for luminal A breast cancer is still controversial (12). At the same time, the reports verified that tumor size was positively correlated with the SLN metastasis rate (13). T1 patients with small tumors and lower SLN metastasis rates (14) are more likely to be exempt from SLNB. So we enrolled 1,619 T1 breast cancer patients in this study and identified predictors for SLN metastasis in T1 breast cancers, especially the relationship between SLN metastasis and molecular subtypes.

The goal of this retrospective study was to establish a predictive model that includes tumor volume, swollen axillary lymph nodes, pathological types, and risk subtypes for SLN metastasis in T1 breast cancers. In addition, patients with a low risk of SLN metastasis could be exempt from SLNB.

## Patients and Methods

### Patients

**Figure 1:**
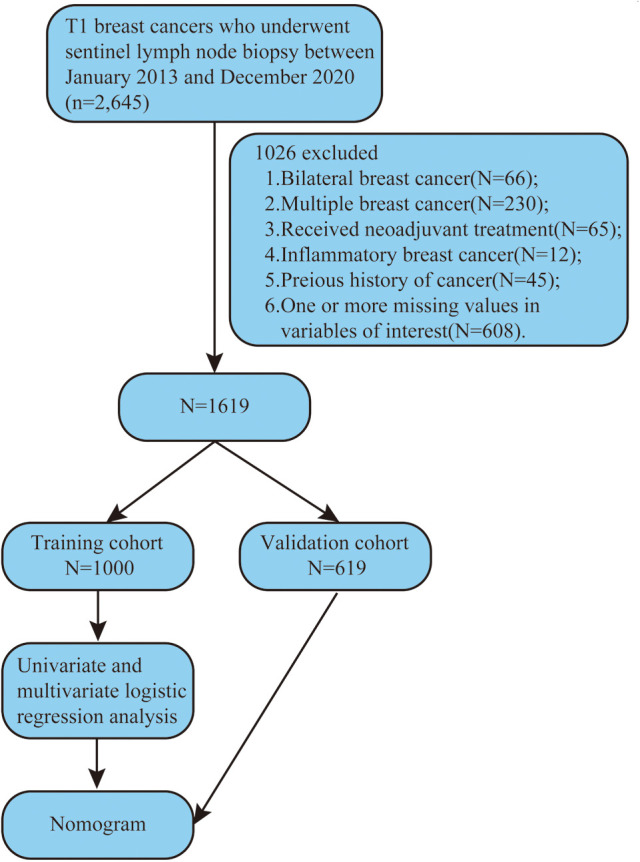
The process for selecting patients for model development.

We reviewed the clinicopathologic data of breast cancer patients with SLN metastasis who underwent SLNB during surgery at Harbin Medical University Cancer Hospital between January 1, 2013 and December 31, 2020. Patients with SLN metastasis were examined by SLNB during surgery. Figure 1 depicts the selection of patients for model development.

### Molecular Typing

Estrogen receptor (ER), progesterone receptor (PR) and ki67 were determined using immunohistochemistry and HER2 by immunohistochemistry or fluorescent *in situ* hybridization (FISH). Based on ER, PR, and HER2 status, patients were categorized into five molecular subtypes: luminal A[ER(+) and/or PR(+), HER2(−), ki67 ≤ 14%]; luminal B HER2(−)[ER(+) and/or PR(+), HER2(−), ki67>14%]; luminal B HER2(+)[ER(+) and/or PR(+), HER2(+)]; HER2 enriched [ER(−) and PR(−), HER2(+)] and triple negative[ER(−) and PR(−), HER2(−)]. Based on univariate analysis results, we regrouped molecular subtypes, and defined them as risk subtypes: low-risk subtype[HER2 enriched]; median risk subtype[Luminal B HER(+) and TNBC]; high-risk subtype[Luminal A and Luminal B HER(−) ].

### Statistical Analysis

Univariate analysis was performed to detect predictors for SLN metastasis. Then, multivariate analysis, including all variables from the univariate analysis that were related to SLN status, was performed to test the factors’ independence. Statistical significance was defined as *p *< 0.05; odds ratio (OR) and 95% confidence intervals (CI) were also calculated. Statistical tests were two-sided, and analyses were performed using SPSS v.19.0 Software (SPSS, Chicago, IL, http://www.spss.com).

## Results

### Clinicopathological and Tumor Anatomical Factors of the Study Population

1,619 female patients with T1 breast cancer were enrolled. 1,000 patients between January 1, 2013 and April 10, 2018 were classified as a training cohort. The remaining 619 patients from April 10, 2018 to December 31, 2020 were classified as a validation cohort. The training cohort and the validation cohort were comparable in clinicopathological and tumor anatomical factors (Table 1). The median patient age was 55 years. The median tumor volume (length × width × width × 0.5) was 936 cm^3^. The SLN metastasis rate of the training cohort was 23.8% (*n* = 1,000), and that of validation was 24.4% (*n* = 619).

**Table 1:** Demographic and baseline characteristics of the study population.

Variables	Total	Training, *N* (%)	Validation, *N* (%)	*P*
No. of cases	1,619	1,000	619	* *
Age				
>55	774	472(47.2)	269(43.5)	0.142
≤55	905	528(52.8)	350(56.5)	
Tumor volume				
>936 cm^3^	611	370(37.0)	234(37.8)	0.745
≤936 cm^3^	1,068	630(63.0)	385(62.2)	
Distance from nipple				
>3 cm	714	418(41.8)	264(42.6)	0.737
≤3 cm	965	582(58.2)	355(57.4)	
Distance from surface				
>6 mm	718	409(40.8)	277(44.7)	0.128
≤6 mm	961	591(59.1)	342(55.3)	
Position of tumor				
Outer upper	710	416(41.6)	271(43.8)	0.502
Upper inner	486	296(29.6)	166(26.8)	
Lower inner	182	115(11.5)	65(10.5)	
Outer upper	301	173(17.3)	117(18.9)	
Swollen lymph nodes				
Positive	454	276(27.6)	170(27.5)	0.952
Negative	1,225	724(72.4)	449(72.5)	
ER				
Positive	1,341	774(77.4)	501(80.9)	0.091
Negative	338	226(22.6)	118(19.1)	
PR				
Positive	1,264	732(73.2)	472(76.3)	0.172
Negative	415	268(26.8)	147(23.7)	
HER2				
Positive	288	190(19.0)	98(15.8)	0.105
Negative	1,391	810(81.0)	521(84.2)	
Ki67				
>14%	752	442(44.2)	278(44.9)	0.780
≤14%	927	558(55.8)	341(55.1)	
Pathological types				
Invasive breast cancer	1,452	854(85.4)	546(88.2)	0.109
Intraductal carcinoma	227	146(14.6)	73(11.8)	* *

*The bold values of P values means a significant difference.*

### The Identification of Independent Prognostic Factors for SLN Metastasis

To determine the independent predictors for SLN metastasis in the training cohort, a univariate analysis was first performed. Only tumor volume and swollen axillary lymph nodes, among tumor anatomical factors, were significantly associated with SLN metastasis (Table 2). Among clinicopathological factors, ER, PR, HER2, pathological types and molecular subtypes were significantly associated with SLN metastasis (Table 3). Therefore, breast cancer patients of ER positive, PR positive, and HER2 negative are more likely to develop SLN metastasis.

**Table 2:** Univariate analysis of tumor anatomical factors.

Variables	No. of positive SLN (%)	OR	95% CI	*P*
Age				
>55	108(22.9)	0.908	0.678–1.217	0.519
≤55	130(24.6)			
Tumor volume				
>936 cm^3^	139(37.6)	3.227	2.390–4.359	**<0**.**001**
≤936 cm^3^	99(15.7)			
Distance from nipple				
>3 cm	104(24.9)	1.107	0.825–1.486	0.497
≤3 cm	134(23.0)			
Distance from surface				
>6 mm	88(21.5)	0.806	0.597–1.088	0.159
≤6 mm	150(25.4)			
Position of tumor				
Outer upper	99(23.8)			
Upper inner	65(22.0)	0.566	0.631–1.286	0.901
Lower inner	27(23.5)	0.943	0.604–1.598	0.982
Outer upper	47(27.2)	0.389	0.798–1.789	1.194
Swollen lymph nodes				
Positive	116(42.0)	3.577	2.629–-4.869	**<0**.**001**
Negative	122(16.9)			* *

*The bold values of P values means a significant difference.*

**Table 3:** Univariate analysis of clinicopathological factors.

Variables	No. of positive SLN (%)	OR	95% CI	*P*
ER				
Positive	214(27.6)	3.216	2.048–5.052	**<0**.**001**
Negative	24(10.6)			
PR				
Positive	205(28.0)	2.770	1.860–4.126	**<0**.**001**
Negative	33(12.3)			
HER2				
Positive	15(7.9)	0.226	0.130–0.391	**<0**.**001**
Negative	223(27.5)			
Ki67				
>14%	108(24.4)	1.065	0.795–1.426	0.675
≤14%	130(23.3)			*** ***
Pathological types				
Invasive breast cancer	235(27.5)	18.096	5.712–57.336	**<0**.**001**
Intraductal carcinoma	3(2.1)			** **
Molecular subtypes				
Luminal A	114(24.1)	4.433	2.002–9.819	**<0**.**001**
Luminal B(HER+)	8(12.3)	1.965	0.677–5.703	*0*.*214*
Luminal B(HER-)	98(39.0)	8.967	3.999–20.110	**<0**.**001**
TNBC	11(10.5)	1.638	0.609–4.405	*0*.*328*
HER2 enriched	7(6.7)			* *

*The bold values of P values means a significant difference.*

**Figure 2:**
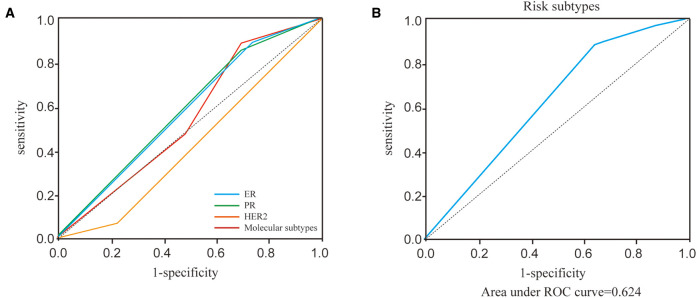
ROC curves of ER, PR, HER2, molecular subtypes and risk subtypes. (**A**) ROC curves of ER, PR, HER2, molecular subtypes. (**B**) ROC curves of risk subtypes.

Before performing multivariate analysis, we analyzed the value of ER, PR, HER2, and molecular subtypes and compared their AUC values through Receiver-operating characteristic (ROC) analysis. The results are shown in Figure 2A and Table 4. The four variables have low AUC values. To improve their AUC, we retyped breast cancer based on the status of ER, PR, and HER2 and defined them as risk subtypes. The AUC value was 0.624 (Figure 2B and Table 4). Furthermore, the univariate analysis also showed that risk subtypes were related to SLN metastasis (Table 5).

**Table 4:** AUC curves of ER, PR, HER2, molecular subtypes and risk subtypes.

Variables	AUC	95% CI	*P*
ER	0.582	0.543–0.621	**<0**.**001**
PR	0.585	0.546–0.624	**<0**.**001**
HER2	0.417	0.378–0.455	**<0**.**001**
Molecular subtypes	0.562	0.524–0.600	**<0**.**001**
Risk subtypes	0.624	0.586–0.661	**<0**.**001**

*The bold values of P values means a significant difference.*

**Table 5:** Univariate analysis of risk subtypes.

Variables	No. of positive SLN (%)	OR	95% CI	*P*
Risk subtypes				
Low risk				
HER2 enriched	7(6.7)			
Median risk				
Luminal B(HER+)	19(10)	1.556	0.632–3.832	*0*.*337*
TNBC				
High risk				
Luminal A	212(30.1)	6.020	2.750–13.179	**<0**.**001**
Luminal B(HER-)				

*The bold values of P values means a significant difference.*

**Figure 3:**
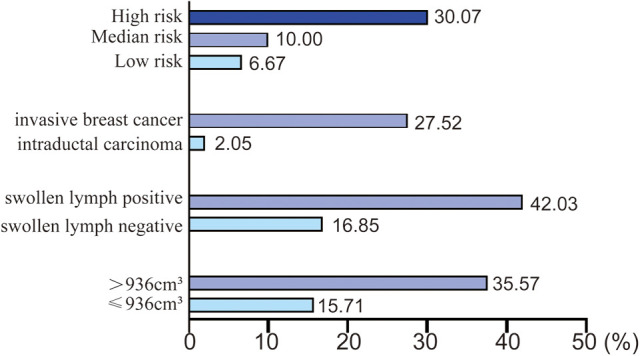
SLN metastasis rate of four independent variables.

Then multivariate analysis indicated that tumor volume, swollen axillary lymph nodes and pathological types were independent statistically significant predictors for SLN metastasis (Table 6). Furthermore, luminal A and luminal B HER2 (−), as the high-risk subtypes, were also independent statistically predictors for SLN metastasis. The SLN metastasis rates of these four variables are shown in Figure 3.

**Table 6:** Multivariate analysis of tumor anatomical location and clinicopathologic variables.

Variables	OR	95% CI	*P*
Tumor volume	5.574	3.382–8.107	**<0**.**001**
Swollen lymph nodes	6.423	4.365–9.453	**<0**.**001**
Pathological types	11.393	3.516–36.917	**<0**.**001**
Risk subtypes			**<0**.**001**
Low risk			**<0**.**001**
Median risk	2.231	0.823–6.048	*0*.*115*
High risk	11.349	4.622–27.868	**<0**.**001**

*The bold values of P values means a significant difference.*

### Construction and Validation of the SLN Metastasis Nomogram

**Figure 4:**
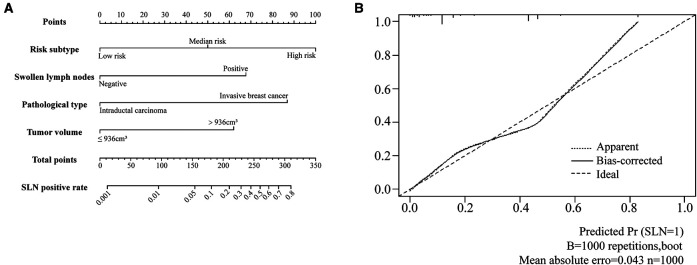
Nomogram to predict the probability of SLN metastases in T1 breast cancer patients and calibration plot. (**A**) The nomogram of SLN metastases rate. (**B**) The calibration plot of nomogram.

The four independent variables, including tumor volume, swollen axillary lymph nodes, pathological types, and risk subtypes, were incorporated to construct the HMU nomogram for estimating the SLN metastasis (Figure 4A). Each factor could be assigned a score by the HMU nomogram (Table 7). By summing the score of each factor together, the total score corresponded to an estimated SLN metastasis rate (Figure 4A).

**Table 7:** Detailed scores of each variable in HMU nomogram.

Variables		Nomogram scores
Tumor volume	>936 cm^3^	62
	≤936 cm^3^	0
Swollen lymph nodes	Positive	68
	Negative	0
Pathological types	Invasive breast cancer	88
	Intraductal carcinoma	0
Risk subtypes	Low risk	0
	Median risk	50
	High risk	100

**Figure 5:**
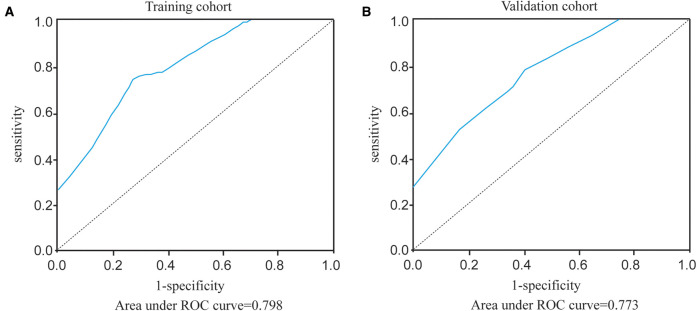
ROC curves of our prediction model in the training cohort and validation cohort. (**A**) Area under ROC curve of training cohort. (**B**) Area under ROC curve of validation cohort.

The constructed HMU nomogram was then validated internally and externally. In the training cohort, ROC analysis showed that the AUC was 0.798 (Figure 5A). When fitted into the validation cohort, the AUC of the prediction model derived from the training cohort was 0.773 (Figure 5B). The calibration curves also revealed that the predictive model could accurately match the SLN metastasis rate (Figure 4B). These results demonstrated that the predictive model performs well in SLN metastasis. For example, the SLN metastasis rate in HER-type intraductal carcinoma, with tumor volume ≤936 cm^3^ and without swollen axillary lymph nodes, is less than 0.1%. We believe that such patients do not require SLNB. If the tumor volume of HER2-invasive breast cancer is ≤936 cm^3^, there is no swollen axillary lymph node. If the SLN metastasis rate is less than 1%, the clinician may not perform SLNB after considering the patient’s wishes and clinical experience. Therefore, by calculating the patient’s SLN metastasis rate according to the above four variables incorporated into the nomogram, we could provide a reference for the patient to decide whether to perform SLNB.

### Patients Exempted from Sentinel Lymph Node Biopsy

**Figure 6:**
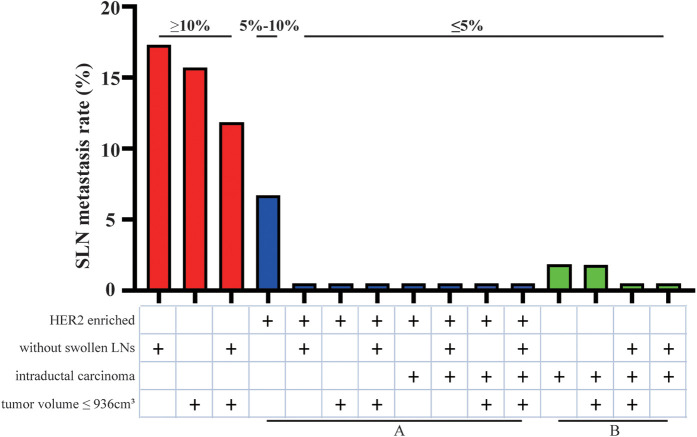
The patients exempted from SLNB.

According to the four variables in the HMU nomogram, we presented the SLN metastasis rate of patients with different characteristics (Figure 6). Patients with low metastasis rates are characterized by intraductal carcinoma (2.05%), low risk (6.67%), and median risk subtypes (10.00%). Therefore, those with HER2 enriched (group A) and intraductal carcinoma (group B) could be included in trials investigating the SLNB exemption. Patients with other characteristics would have lower metastasis rates, such as those with HER2 enriched associated tumor volume smaller than 936 cm^3^ or without axillary lymphadenopathy, so they also could be included in this study.

## Discussion

The SLN metastasis is the gold standard for assessing ALN metastasis, but SLNB still has the following problems: positive SLN exemption, false negative rate, and complications after SLNB (6–9). Therefore, patients could avoid SLNB if some screening criteria can be defined to correctly assess the sentinel metastasis.

To fully evaluate the tumor size in this study, we adopted the concept of tumor volume, which took into account the tumor’s long diameter and short diameter. When the tumor volume is less than or equal to 936 cm^3^, the SLN metastasis rate is low (15.71%). This is consistent with previous studies that large tumors increase the risk of SLN metastasis (15–17). Swollen axillary lymph nodes are also highly suggestive of SLN metastasis (42.03%). However, some lymph node enlargement without SLN metastasis may be caused by congenital development of inflammation (18).

There is still controversy about whether SLNB should be performed in ductal carcinoma of breast cancer (19, 20). According to a meta-analysis, the incidence of SLN metastasis was 7.4 in patients with a preoperative diagnosis of intraductal carcinoma (21). Another study suggested that the only criterion for recommending SLNB in intraductal carcinoma should be any uncertainty about the presence of invasive lesions (22). Therefore, considering the risk of missed detection of microinvasion in some intraductal carcinomas and the high risk of intraductal carcinomas, we included intraductal carcinomas in the study. Intraductal carcinoma of the high-risk subtype has a tumor volume greater than 936 cm^3^, accompanied by swollen axillary lymph nodes, and the SLN metastasis rate is as high as 30%, so SLNB should be performed. Studies have shown that the positive rate of SLNB in patients diagnosed with intraductal carcinoma by preoperative core needle biopsy is significantly higher than that in patients diagnosed with intraductal carcinoma after surgery (23, 24). If the preoperative diagnosis of intraductal carcinoma with swollen axillary lymph nodes is associated with undetected microinvasion, core needle biopsy should be performed to confirm the status of the swollen axillary lymph nodes (25–27).

Moreover, among the five molecular types of breast cancer, luminal A and luminal B HER2(−) have the highest SLN metastasis rate (30.07%). In other words, patients with ER(+)/PR(+)/HER2(−) T1 breast cancer are more likely to develop SLN metastasis. This is also consistent with previous studies, which confirm that triple-positive breast cancer is more prone to SLN metastasis (28), and that triple-negative breast cancer has a lower SLN metastasis rate (29). Our study demonstrated that ki67 has no effect on SLN metastasis of T1 breast cancer, which is consistent with Fabinshy's finding (30). However, another study found that ki67 was positively correlated with SLN metastasis (31). T1 breast cancer may be smaller, on the other hand, so ki67 is more likely to reflect the proliferation state rather than metastasis.

According to the study on an American breast cancer patient conducted by Memorial Sloan Kettering Cancer Center (MSKCC), age, tumor size, tumor type, lymphovascular invasion, tumor location, multifocality, ER and PR were all associated with SLN metastasis (32). The nomogram’s AUC is 0.754. The Fudan University Shanghai Cancer Center in China, with an AUC value of 0.7649, included age, tumor size, tumor location, tumor type, and lymphovascular invasion (33). Two studies predicted the risk factors of SLN metastasis, but they ignored the impact of molecular subtypes on SLN metastasis. More importantly, our study focused on patients with low SLN metastasis rate. We thought that T1 breast cancer patients reduced the implementation of SLNB with less risk. The AUC value is 0.798 in the HMU nomogram, indicating that SLNB could be avoided more safely and effectively.

In conclusion, we developed and validated a nomogram for predicting SLN metastasis by adopting clinicopathological and tumor anatomical factors location from 1,000 T1 breast cancer patients. The remaining 619 T1 breast cancer patients were classified as validation cohort for external validation. The HMU nomogram provides comprehensive SLN metastasis information to optimize surgical procedures and benefit breast cancer patients. We focused on patients included in the SLNB exemption study, including intraductal carcinoma, HER2-enriched. Those with HER2-enriched and other low-risk factors may also be included in the study.

The potential limitations should be considered. First, more patients’ information from other hospitals will be more useful for validating HMU nomograms. Second, the SLNB exemption only applies to T1 breast cancer patients, and additional and refined HMU nomograms should be further studied for various types of breast cancer patients.

## Data Availability

The raw data supporting the conclusions of this article will be made available by the authors, without undue reservation.
